# Acceptability and Usability of a Digital Medicines Tool for Dental Practitioners in four Southeast Asian Countries

**DOI:** 10.1016/j.identj.2025.109361

**Published:** 2026-01-14

**Authors:** Leanne Teoh, Ruby Biezen, Marietta Taylor, Nadia Kaunein, Wendy Thompson, Charlene Goh, Jacob Chew, Yuniardini Septorini Wimardhani, Mohd Hafiz Arzmi, Wan NurHazirah Wan Ahmad Kamil, Sireerat Sooampon, Benjar Issaranggun Na Ayuthaya, Michael McCullough

**Affiliations:** a Melbourne Dental School, University of Melbourne, Melbourne, Australia; b Department of General Practice, University of Melbourne, Melbourne, Australia; c Melbourne School of Population and Global Health, University of Melbourne, Melbourne, Australia; d Division of Dentistry, School of Medical Sciences, Faculty of Biology, Medicine and Health, University of Manchester, Manchester, UK; e Faculty of Dental Medicine, Universitas Airlangga, Surabaya, Indonesia; f Faculty of Dentistry, National University of Singapore, Singapore; g Department of Oral Medicine, Faculty of Dentistry, Universitas Indonesia, Jakarta, Indonesia; h Kulliyyah of Dentistry, International Islamic University Malaysia; i Faculty of Dentistry, University Teknologi MARA (UiTM), Selangor, Malaysia; j Department of Pharmacology, Faculty of Dentistry, Chulalongkorn University, Bangkok, Thailand

**Keywords:** Digital health, Clinical decision tool, Dental prescribing, Dental antibiotic, Antibiotic resistance

## Abstract

**Introduction and aims:**

Antimicrobial resistance is a global public health emergency and a prominent issue in Southeast Asia. There is also a lack of dental-specific drug resources to assist with medication management for dentists. Our dental-specific decision support tool, MIMS Drugs4dent, was developed to assist Australian dentists optimize their prescribing. The aim of this study was to trial MIMS Drugs4dent for acceptability and usability with dental practitioners in Indonesia, Malaysia, Singapore, and Thailand.

**Methods:**

This was a cross-sectional study of MIMS Drugs4dent with dental practitioners in Indonesia, Malaysia, Singapore, and Thailand. Dental practitioners were provided access to MIMS Drugs4dent for 1 month. After the intervention period, participants completed an online survey that assessed their perceptions of the tool. The primary outcome was the acceptability and usability of MIMS Drugs4dent, using the Framework for Acceptability and System Usability Scale, respectively. The secondary outcome was to determine the resources used by dental practitioners for drug information. Descriptive statistics and thematic analysis were used for quantitative and qualitative data analysis, respectively.

**Results:**

From January to June 2025, 30 dentists from each country participated. Most participants (93.4% and 94.2%) agreed or strongly agreed that MIMS Drugs4dent can improve their ability to prescribe according to guidelines and access dental-relevant drug information, respectively. Most participants (88.3% and 82.5%) agreed or strongly agreed that MIMS Drugs4dent was easy to use and that information could be located quickly. However, participants preferred a local drug database and a mobile-rendered version to improve usability. A wide range of sources (n = 62) were reported to be used for medicines information.

**Conclusion:**

MIMS Drugs4dent had high acceptability and usability in this study. With adaptation using country-specific localised drug databases, the tool has potential for use in dentistry in these countries. The study underscores the importance of including dentistry in broader digital health strategies.

## Introduction

Antimicrobial resistance (AMR) is a significant global public health issue, being attributable to 1.27 million deaths worldwide in 2019.^1^ AMR is driven by antimicrobial use. Risk assessments by the World Health Organization (WHO) have shown that the Southeast Asian region is likely the most at-risk part of the world.^2^, ^3^, ^4^ In dentistry, antibiotic prescribing contributes to approximately 10% of all prescribed antibiotics across human health care worldwide,^5^ with up to 80% being inappropriate.^6^^,^^7^

Several international studies of prescribing practices, including in Southeast Asia, demonstrate inappropriate prescribing by dental practitioners. Prescribing for noninfective conditions, localised infections where dental treatment alone would be sufficient, and other non-clinical reasons, such as patient expectations for antibiotics, all occur in dental practice.^8^^,^^9^ Due to the lack of surveillance in these countries, there are no robust data on dispensed antibiotics prescribed by dental practitioners.^2^ Tracking of antibiotic usage is further complicated by the availability of antibiotics from pharmacies with or without a prescription in some countries in this region.^10^ Other challenges exist in this region, including poor access to oral care and fragmented health information systems.^11^ Furthermore, there is a lack of dental-specific drug resources to assist with medication management and prescribing for dentists in Southeast Asia. While Indonesia, Malaysia, and Singapore have some dental prescribing guidelines,^12^, ^13^, ^14^ they do not aid with the perioperative medication management of patients in dental practice.

The WHO has developed the “Action Plan for Oral Health in South-East Asia 2022-2030” and the “WHO South-East Asia Regional Roadmap for Results and Resilience”. Both plans have highlighted the issue of AMR.^3^^,^^4^ and include recommended strategies to improve oral health care. One such strategy is to use digital technologies to enhance research and dental practice, as well as assist with tackling AMR.^3^^,^^4^^,^^15^ Digital medicine support tools are increasingly implemented throughout health care to provide timely access to drug information. They can also help clinicians by providing interventions to help with safe prescribing, such as avoiding drug and allergy interactions.

In Australia, there is no source of drug compendia specific to dentistry. To assist with access to relevant drug information and prescribing in Australia, our team has developed and trialed a medicines decision support tool for Australian dentistry, Drugs4dent.^16^ Drugs4dent provides timely, dental-relevant drug information, patient education on appropriate antibiotic use, and assists dentists to prescribe according to Australian dental guidelines.^16^ Drugs4dent, which has been trialed in a before-and-after pilot study, was associated with a 41% reduction in the number of prescribed antibiotics.^16^ It has been shown to have high acceptability and usability with Australian dentists.^17^ This tool was commercialised with MIMS Australia in 2024 to produce MIMS Drugs4dent.^18^ MIMS Australia (originally known as the Monthly Index of Medical Specialities) is a leading supplier of drug compendia in Australia and New Zealand. MIMS is also a leading supplier of drug compendia in Asia and the Asia Pacific.

Our intervention of MIMS Drugs4dent directly addresses one of the strategic action plans by the WHO, by providing digital innovations to assist with strengthening oral health care.^3^ It also addresses the challenge of tackling AMR and integration of strategies to improve antibiotic prescribing.^3^ The WHO has specific member states as part of the Southeast Asian region, although geographically, countries including Indonesia, Malaysia, Singapore, and Thailand are all located in Southeast Asia. One challenge facing the dental profession in Southeast Asia is the lack of dental-specific drug resources. While MIMS Drugs4dent uses an Australian drug database, we wanted to gain insight into the acceptability and usability of this product in Southeast Asia, to inform future possible adaptation of this product for different countries. Thus, the aim of this study was to assess the acceptability and usability of MIMS Drugs4dent for dentistry in 4 countries in Southeast Asia: Indonesia, Malaysia, Singapore, and Thailand.

## Methods

### Study design and participants

This research employed a postintervention cross-sectional study design to evaluate the acceptability and usability of MIMS Drugs4dent among dental professionals across 4 countries in Southeast Asia. The study aimed to recruit a total of 120 dentists, with 30 participants from each of the following nations: Indonesia, Malaysia, Singapore, and Thailand. Efforts were made to ensure a diverse and representative sample by considering factors such as gender and years of clinical experience. This study was reported according to the Checklist for Reporting of Survey Studies (CROSS) reporting guideline.^19^ The CROSS checklist is shown in Supplementary Table 1.

### Ethical approval

Ethical approval was obtained from the Office of Research Ethics and Integrity at the University of Melbourne (ID: 2024-31143-60007-3; 2025-31145-63819-6), the Research Ethics Committee at the Faculty of Dentistry Universitas Indonesia (ID: 139/Ethical Approval/FKGUI/II/2025), the International Islamic University Malaysia Research Ethics Committee (ID: IREC 2025-018), the Institutional Review Board at the National University of Singapore (ID: NUS-IRB-2024-753), and the Human Research Ethics Committee of the Faculty of Dentistry, Chulalongkorn University (ID: HREC-DCU2025-024).

### Recruitment process and procedures

The selection of participants was facilitated through the professional dental networks in these countries by using convenience sampling. Potential participants were invited to take part in the study via email communication. An initial recruitment email was sent, together with a plain language statement. Follow-up emails were sent every 2 weeks, to a maximum of 3 emails.

Prior to commencing the study, written informed consent was obtained from all participants. The dentists were provided with access to MIMS Drugs4dent for 1 month. Instructions were provided on how to use the tool, which could be used either in clinical practice with patients or for independent exploration.

At the end of the trial, participants were asked to complete an acceptability and usability survey assessing their perceptions of MIMS Drugs4dent within the context of their country’s dental practices. The survey was conducted online using Qualtrics and was based on the Framework for Acceptability and System Usability Scale.^20^^,^^21^ It comprised 5 sections (Demographics, About MIMS Drugs4dent, Acceptability, Usability, and Clinical Workflow) and consisted of 30 questions (Supplementary Figure 1). The survey was piloted with 5 dental practitioners from Singapore; no modifications were needed. Participants were provided with an individual link to the survey and sent an email reminder if they had not completed the survey in 2 weeks. The functionality to “prevent multiple submissions” was activated to allow only 1 survey entry per participant. In appreciation of their time, each participant received a $50 AUD grocery voucher upon completion of the study.

### Intervention

**Figure 1:**
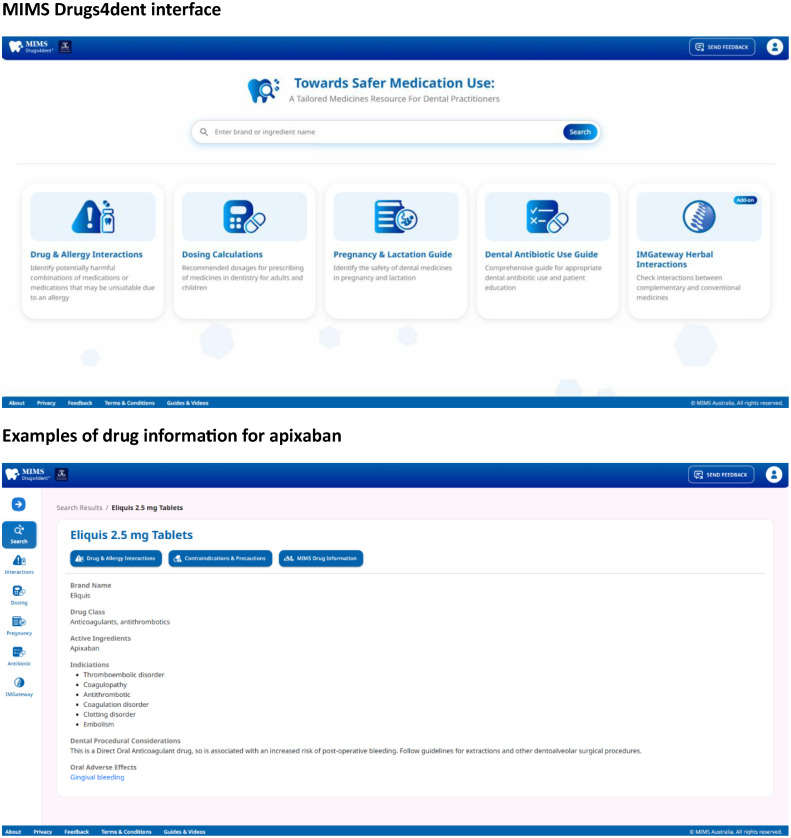

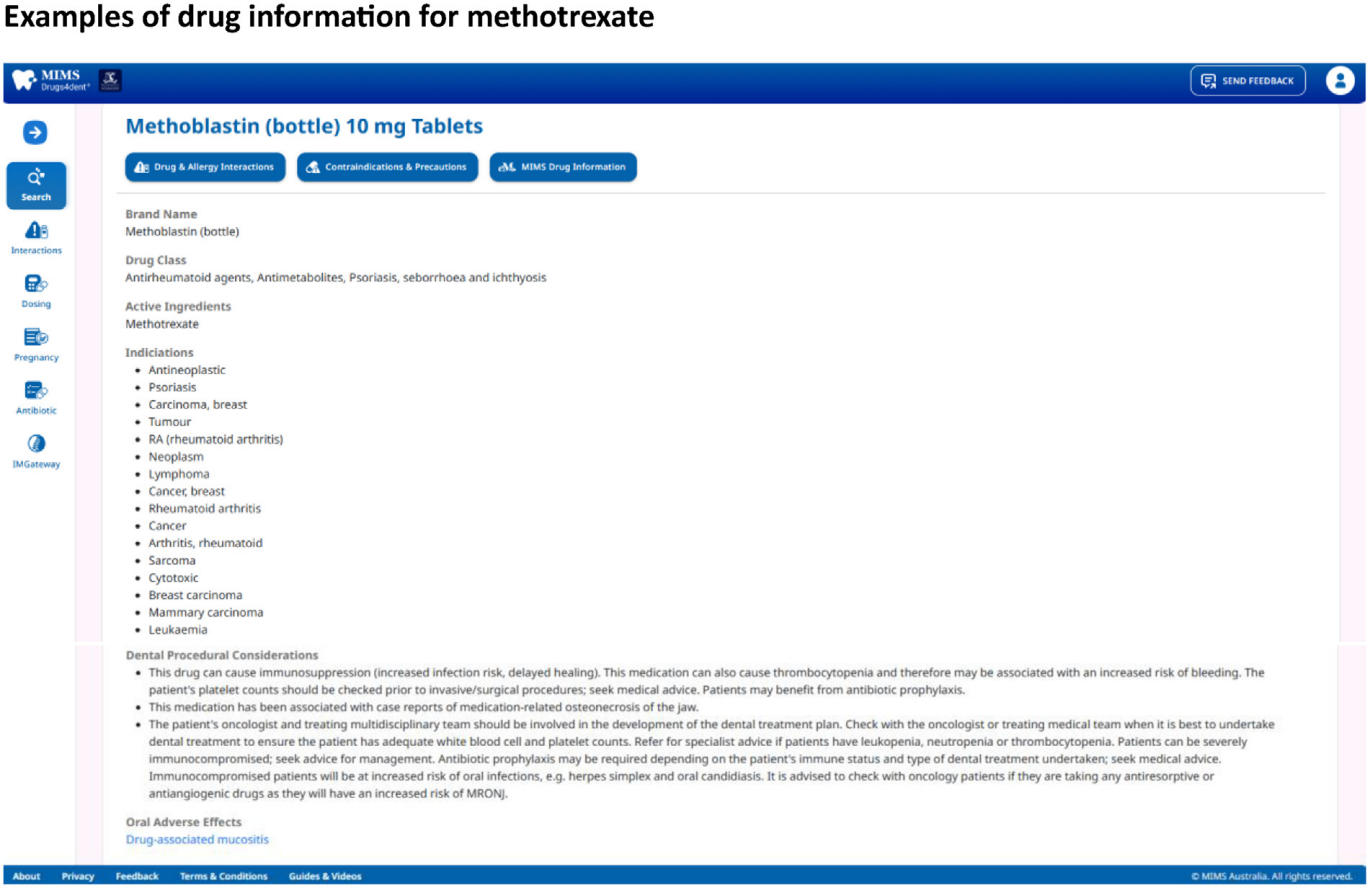
MIMS Drugs4dent interface

**Figure 2:**
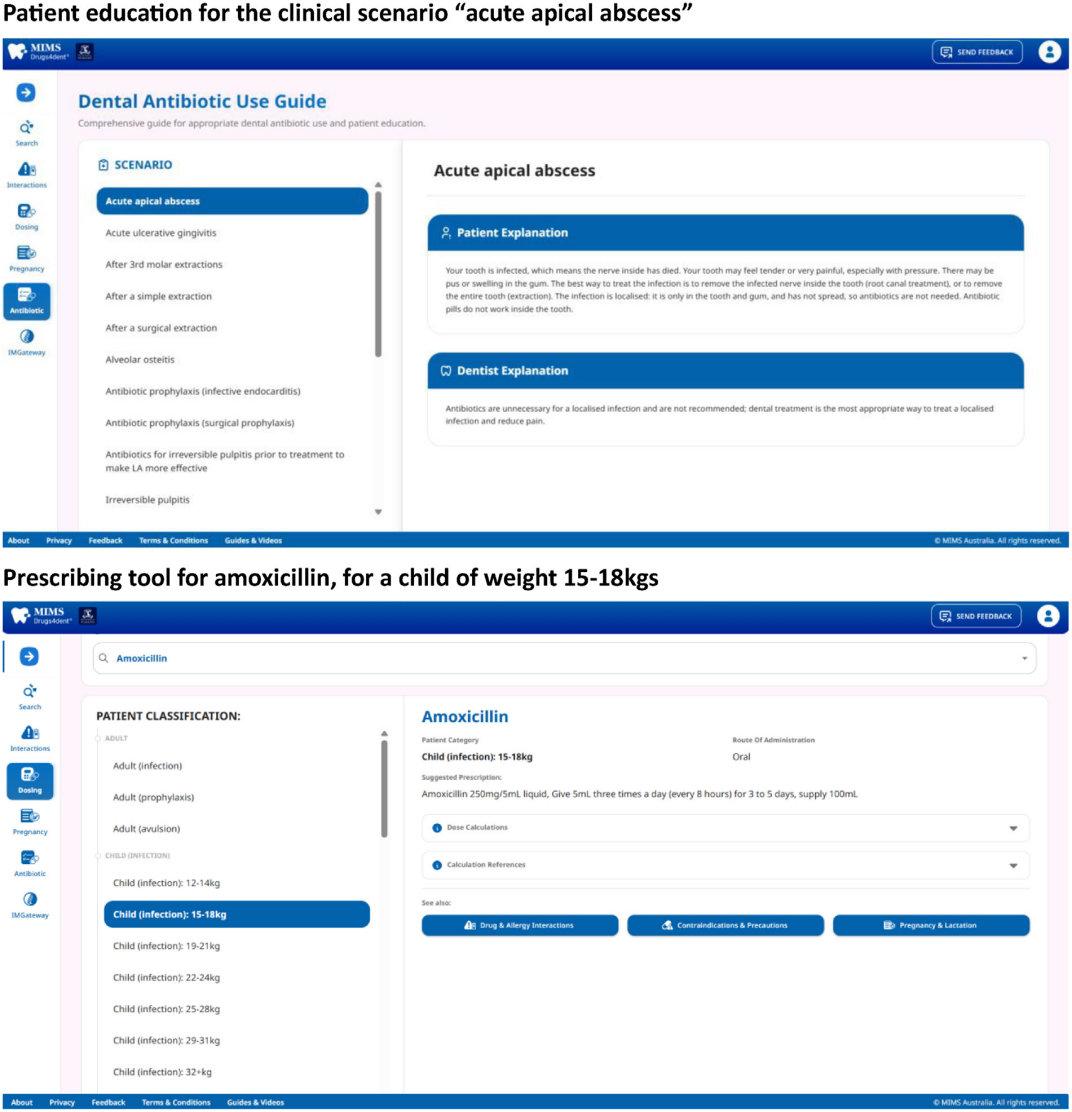
Patient education for the clinical scenario “acute apical abscess” and prescribing tool for amoxicillin for a child weighing 15 to 18 kg.

MIMS Drugs4dent^18^ is a standalone, medicines decision support tool designed for dental practice. Novel features of MIMS Drugs4dent that are not readily available in other Australian drug stores include dental procedural considerations and education for patients and dental practitioners on the appropriate use of antibiotics in dentistry. Dental procedural considerations include drugs associated with medication-related osteonecrosis of the jaw or those that contribute to an increased risk of bleeding. Examples of the drugs apixaban and methotrexate in the MIMS Drugs4dent user interface are shown in Figure 1. Additionally, MIMS Drugs4dent incorporates a prescribing tool to assist dentists in prescribing according to the Australian national prescribing guidelines.^22^ Finally, the tool provides safety checks for drug-drug, drug-disease, and drug-allergy interactions, and information on medication safety during pregnancy and lactation. Figure 2 shows examples of patient education and the prescribing tool in the MIMS Drugs4dent user interface.

### Outcomes and data analysis

The primary outcome was the acceptability and usability of MIMS Drugs4dent, assessed through the Framework for Acceptability and the System Usability Scale.^20^^,^^21^ Acceptability is defined as “a multi-faceted construct that reflects the extent to which people delivering or receiving a healthcare intervention consider it to be appropriate, based on anticipated or experienced cognitive and emotional responses to the intervention.”^23^ Usability is defined as the evaluation of a digital system to assess how easily users can interact with the interface.^24^ Definitions include attributes such as learnability, efficiency, and error recovery.^24^ Additionally, the study aimed to investigate the types of resources currently used by dental practitioners in the region to guide the medication management of their patients.

Descriptive statistical analyses were used to analyse the survey data. Likert scales were used to capture participants’ responses related to acceptability and usability, with the information presented by category and summarised using proportions.

For the qualitative component, 2 researchers (L.T. and R.B.) independently coded the participants’ responses to identify relevant concepts and ideas pertaining to acceptability, usability, and recommendations for improving the tool. Deductive thematic analysis was conducted, with a coding scheme derived during the data collection process and based on a previous similar pilot study.^17^ The researchers then consolidated and refined their coding schemes by merging codes to form overarching themes and subthemes, resolving any discrepancies through discussion and consensus. The qualitative data were managed and organised using NVivo software (QSR International).

### Role of the funding source

The funder of this study had no role in study design, data collection, data analysis, data interpretation, or writing of the report.

## Results

### Demographics

A total of 145 dental practitioners were invited to participate in the study. Recruitment ceased when 120 consented, comprising 30 dentists from each country.

Participants were predominantly female (69.2%) and worked in public practice (73.7%). Most had 10 years or less of clinical experience (57.6%). General practitioners comprised 66.1% of participants, with the remaining being dental specialists (33.9%). Oral and maxillofacial surgeons and oral medicine specialists were the most common specialties reported (57.5% of all specialties) (Table 1).

**Table 1:** Demographic characteristics of surveyed dental practitioners.

Characteristic	No. (%)
Sex	
Male	36 (30.0)
Female	83 (69.2)
Other	1 (<1.0)
Years of experience	
≤10 y	68 (57.6)
11 to 20 y	35 (29.7)
21 to 30 y	15 (12.7)
Main practice sector	
Private practice	31 (26.3)
Public sector	87 (73.7)
Speciality	
General dental practitioner	78 (66.1)
Specialist	40 (33.9)
Specialist dentists	40 (100.0)
Paediatrics dentistry	5 (12.5)
Periodontics	3 (7.5)
Orthodontics	2 (5.0)
Endodontics	3 (7.5)
Prosthodontics	1 (2.5)
Oral maxillofacial surgery/oral medicine	23 (57.5)
Other*	3 (7.5)

⁎ Other: Special care dentistry and pathologist.

### Acceptability

Most participants (93.3%, n = 112) agreed or strongly agreed that MIMS Drugs4dent was acceptable. Most (94.2%, n = 113) agreed or strongly agreed that the tool could improve their ability to access dental-relevant drug information, thereby improving patient management. Of the participants, 95.8% agreed or strongly agreed that MIMS Drugs4dent could improve their ability to prescribe appropriately and safely with regard to drug and allergy interactions, pregnancy, and breastfeeding considerations. Most participants (93.4%, n = 112) also agreed or strongly agreed that MIMS Drugs4dent could improve their ability to prescribe the appropriate drug regimen and realised the benefit in having dental-specific drug information (Table 2).

“It is a valuable resource, especially for identifying oral implications of medications.”

(Indonesia, ≤10 years’ experience, private practice)

“The MIMS system is a step in the right direction in ensuring safe and effective prescribing . . . rather than go through an unreliable google search.”

(Singapore, ≤10 years’ experience, public practice)

**Table 2:** Acceptability, usability, and user experience of MIMS Drugs4dent.

ACCEPTABILITY				
		Comfortable or very comfortable	Neither agree nor disagree	Uncomfortable or very uncomfortable
How comfortable did you feel using Drugs4dent?		94 (78.3)	13 (10.8)	13 (10.8)
		Little or no effort	Neither agree nor disagree	A lot, or a huge effort
How much effort did it take to use Drugs4dent?		83 (79.2)	25 (20.8)	12 (10.0)
		Agree or strongly agree	Neither agree nor disagree	Disagree or strongly disagree
Drugs4dent can improve my ability to access dental-relevant drug knowledge to assist with improved patient management regarding medication use.		113 (94.2)	5 (4.2)	2 (1.6)
Drugs4dent can improve my ability to prescribe appropriately, regarding regimen (dose, duration, and frequency), and according to guidelines.		112 (93.4)	6 (5.0)	2 (1.6)
Drugs4dent can improve my ability to prescribe appropriately and safely with respect to drug and allergy interactions, pregnancy, and breastfeeding considerations.		115 (95.8)	4 (3.3)	1 (0.8)
It is clear to me how Drugs4dent will help me access dental-relevant drug knowledge and prescribe more safely.		113 (94.2)	6 (5.0)	1 (0.8)
Engaging with Drugs4dent chairside would interfere with my workflow.		28 (23.3)	29 (24.2)	63 (52.5)
		Confident or very confident	Neither confident nor unconfident	
How confident did you feel about using Drugs4dent?		108 (90.0)	12 (10.0)	
		Acceptable or completely acceptable	Neither acceptable nor unacceptable	
How acceptable was MIMS Drugs4dent?		112 (93.3)	8 (6.7)	
USABILITY		Agree or strongly agree	Neither agree nor disagree	Disagree or strongly disagree
Overall, I found it easy to use MIMS Drugs4dent.		106 (88.4)	12 (10.0)	2 (1.7)
I was able to access information I need quickly using MIMS Drugs4dent.		99 (82.5)	15 (12.5)	6 (5.0)
It was easy to learn to use MIMS Drugs4dent.		114 (95.0)	6 (5.0)	
It was easy for me to find the information I needed in MIMS Drugs4dent.		106 (88.4)	10 (8.3)	4 (3.3)
Whenever I made a mistake using MIMS Drugs4dent, I could recover easily and quickly.		101 (84.2)	15 (12.5)	4 (3.3)
FORMAT AND USER EXPERIENCE		Agree or strongly agree	Neither agree nor disagree	Disagree or strongly disagree
It was easy for me to find the information I needed in MIMS Drugs4dent.		106 (88.4)	10 (8.3)	4 (3.3)
The interface of MIMS Drugs4dent was pleasant.		107 (89.2)	9 (7.5)	4 (3.3)
MIMS Drugs4dent has all the functions and capabilities I need for accessing drug knowledge and appropriate prescribing.		106 (88.4)	9 (7.5)	5 (4.1)
Overall, I am satisfied with MIMS Drugs4dent.		110 (92.0)	7 (5.8)	3 (2.5)
MIMS Drugs4dent is an important and helpful tool in clinical practice, to help with safe prescribing and appropriate use of medicines in dentistry.		107 (89.2)	11 (9.2)	2 (1.7)
I would like to have MIMS Drugs4dent available to me for clinical practice dentistry.		111 (92.5)	8 (6.7)	1 (0.8)
CLINICAL WORKFLOW		Agree or strongly agree	Neither agree nor disagree	Disagree or strongly disagree
The clinical workflow using Drugs4dent was clear.		113 (94.2)	6 (5.0)	1 (0.8)
		No, I prefer Drugs4dent as a standalone system	Yes, I think integration would be better	
Would you prefer the tool integrated into your dental practice software (linked to individual patients, with patient information and prescriptions stored in your dental practice management software), or do you prefer it as a standalone system?		39 (32.5)	81 (67.5)	
	Once <5 patients	Once every 5-10 patients	Once every 10-20 patients	Once >20 patients
Now that you have used MIMS Drugs4dent, how often do you anticipate that you would use this tool?	24 (20.0)	40 (33.3)	41 (34.2)	15 (12.5)

Values are presented as number (%).

Surveyed dental practitioners felt both comfortable and confident using the program, with 94.2% (n = 113) reporting it was clear how MIMS Drugs4dent would help them access drug knowledge. Most participants (93.3%, n = 112) agreed or strongly agreed that the tool was acceptable and especially helpful for patients taking multiple medications (Table 2).

“MIMS Drugs4dent is an excellent initiative that bridges the gap between pharmacology and dental practice. Its evidence-based content, intuitive interface, and practical guidance make it a valuable tool in both general and complex clinical scenarios.”

(Indonesia, 11-20 years’ experience, public practice)

*“*Very comprehensive and appreciate the oral side effects stated too. Helpful for a special care dentist treating medically compromised patients.”

(Malaysia, 11-20 years’ experience, public practice)

“I think MIMS made me more confident with my medication for my patients.”

(Indonesia, ≤10 years’ experience, private practice)

Furthermore, dental practitioners recognised the value of readily available guidelines to aid their decision-making when prescribing.

“It also supports evidence-based practice and ensures that treatment choices are aligned with current guidelines.”

(Malaysia, ≤10 years’ experience, private practice)

Dental practitioners recognised some different medications were contained in an Australian drug compendium, and a recommendation was that the drugs listed be included in their local drug database.

“However, a key area for improvement, particularly for users in Indonesia, is the comprehensiveness of its local drug database . . . we’ve encountered instances where certain drugs . . . commonly prescribed in Indonesia are not listed.”

(Indonesia, ≤10 years’ experience, public practice)

Practitioners expressed different opinions on whether MIMS Drugs4dent should reference Australian or local guidelines. Some antibiotics prescribed in these countries by dental practitioners were different to Australia, and not all these were included in MIMS Drugs4dent. Furthermore, paediatric drug regimens differed compared to Australia, which was reflected in feedback.

“The current methods for calculating children’s drug dosages have limitations and do not cover all medications.”

(Thailand, 21-30 years’ experience, public practice)

“I would love to see even more localized guidelines”

(Thailand, ≤10 years’ experience, private practice)

### Usability

Most participants (88.4%, n = 106) agreed that MIMS Drugs4dent was easy to use, and 82.5% (n = 99) could locate required information quickly (see Table 2).

“It was a useful tool, was easy to navigate, and is user friendly.”

(Singapore, ≤10 years’ experience, public practice)

Respondents found the interface pleasant and the clinical workflow clear (94.2%, n = 113). Furthermore, the potential for its use in teaching has also been highlighted.

“Helpful tool in clinical practice as well as potential to assist in teaching and learning activities.”

(Malaysia, 11-20 years’ experience, public practice)

However, 23.3% (n = 28) indicated that MIMS Drugs4dent would interfere with their clinical workflow. Most respondents indicated that they would use MIMS Drugs4dent on their mobile phone, as this was how it was mostly accessed during clinical practice.

“While the interface functions well on a computer, the mobile web version presents redundant layers within each window. It may be beneficial . . . to enhance user experience on mobile devices.”

(Thailand, ≤10 years’ experience, public practice)

“Make it an app for easy access, patient explanation part is useful!”

(Singapore, ≤10 years’ experience, public practice)

### Medicine resources

A wide range of guidelines and other resources was reported to be used by dental practitioners to guide the perioperative management of patients and prescribing. Sixty-two different resources were cited for use across all countries. Participants reported using between 1 and 6 different resources. The most common individual source cited by survey participants was “google” (39.2%, n = 47), followed by the American Heart Association guidelines (35.8%, n = 43) and the American Dental Association guidelines (27.5%, n = 33) (Table 3).

**Table 3:** The most common references cited by participants as resources for drug information (N = 120).

Sources	n	%
Google*	47	39.2
American Heart Association guidelines	43	35.8
American Dental Association guidelines	33	27.5
MIMS†	20	16.7
Formulari Ubat KKM Blue Book Ministry of Health Malaysia	8	6.7
National Antimicrobial Guidelines Ministry of Health Malaysia	6	5.0
Textbooks and school notes	6	5.0
American Association of Endodontics Guidance on the Use of Systemic Antibiotics in Endodontics	4	3.3
National Antimicrobial Guidelines	4	3.3
Rational Drug Use in Dentistry Faculty of Dentistry Chulalongkorn University	4	3.3
American Academy of Pediatric Dentistry guidelines	3	2.5
Journals	3	2.5

⁎ Google search and Google Scholar.

† MIMS and MIMS Drugs4dent.

Some country-specific guidelines were cited (eg, Blue Book Ministry of Health Malaysia and Rational Drug Use in Dentistry Faculty of Dentistry Chulalongkorn University). Some other resources were cited from other countries, such as the American Dental Association guidelines, or did not apply to dentistry, such as the WHO analgesic pain ladder. Supplementary Table 2 shows all the resources cited by participants for drug information.

## Discussion

To our knowledge, this is the first reported study evaluating a dental medicines decision tool in Southeast Asia. The high acceptability of the tool and agreement that MIMS Drugs4dent can assist with safe and appropriate prescribing and locating dental-relevant drug information, suggests that this tool can help provide access to evidence-based medication knowledge for dentistry. There were numerous and varied resources used by dental practitioners for drug information in the current study, with differing levels of evidence. This potentially reflects the lack of comprehensive local guidance and the challenge in accessing dental-relevant drug information for these dental practitioners.

In recent years, there has been a surge of digital health initiatives in the Southeast Asian region. These include telemedicine interventions and big data analytics for predicting future health demands.^25^ There has been rapid piloting and adoption of a range of digital health technologies in Southeast Asia, which include repurposing established digital solutions.^25^ Digital health interventions can also be used for oral cancer screening and early detection.^26^ Dentistry has also seen an uptake in clinical decision tools, including those that assist in managing paediatric dental trauma and caries.^27^^,^^28^ Digital tools to reduce inappropriate opioid prescribing have also been trialed in dentistry.^29^ In addition, there has been an increase in the use of teledentistry to enable the provision of some types of dental treatment from distant locations.^30^ Given that most medicines information is currently accessed through Internet sources, it suggests that dentists are willing to access e-tools for drug knowledge. Incorporation of these tools as part of electronic medical records has been shown to increase uptake.^31^ This study has shown that with adaptation, MIMS Drugs4dent has the potential to be acceptable and usable in these countries, although there are discrepancies with the digital health maturity in dentistry in Southeast Asia.^11^ In this present study, some public institutions have Internet segregation to reduce access to untrusted networks.^32^ Thus, dental practitioners tended to access MIMS Drugs4dent through their mobile phones. In Australia, dentists usually access the program through a computer, and at the time of this study, MIMS Drugs4dent did not have a mobile-rendered application. This accounts for some of the feedback received in this study. Regardless of the digital health maturity of each country, access to smartphones is universal. Future research can be directed toward trialling MIMS Drugs4dent as a standalone, mobile-rendered application. This may enable timely and easy access to evidence-based, dental-specific drug information to assist with patient management and appropriate prescribing.

While there was high acceptability and usability of MIMS Drugs4dent in this present study, several system-level barriers to implementation potentially exist. Regulatory requirements and compliance need to be accounted for in each country, while ensuring a user-centered design.^33^^,^^34^ This also includes data privacy concerns,^34^ although MIMS Drugs4dent does not store any patient data and solely provides dental-specific drug information. MIMS Drugs4dent would also require adaptation using the respective country’s local drug compendia and guidelines, to ensure contextualization of the information. Ethical considerations in producing a drug compendium also need to be considered, including medicolegal responsibility for the information provided, as well as the cost viability of the product. MIMS is already a leading supplier of drug compendia for health care professionals throughout Asia Pacific. However, involvement with end users and considering all potential factors that affect implementation are essential for future research regarding the development and design of the decision tool.

WHO risk assessments for AMR have shown that the Southeast Asian region is possibly the most at-risk region in the world.^2^, ^3^, ^4^ Approximately 30% of deaths attributable to AMR occur in this area.^2^ In 2011, Health Ministers from SEA adopted the Jaipur Declaration for Prevention and Control of AMR to commit to tackling this public health issue.^35^ Addressing AMR in this region is complicated by other factors such as loosely regulated access, supply, and availability of antibiotics; self-medication by patients; and the availability of substandard or counterfeit antibiotics.^2^^,^^10^ Dental antibiotic prescribing accounts for about 10% of all prescribed antibiotics worldwide.^5^ There are no published data on dental antibiotic consumption by countries in this region. However, inappropriate antibiotic prescribing is common in dentistry worldwide, with up to 80% inappropriate prescribing by dentists, for both therapeutic and prophylactic reasons.^6^^,^^7^ One action plan by the WHO to assist with AMR in Southeast Asia includes the use of digital tools and technologies.^3^^,^^4^ Partly due to the inclusion of patient education about appropriate antibiotic use and prescribing recommendations, Drugs4dent was associated with a 41% reduction in dental antibiotic prescribing in a pre-post pilot study.^16^ Decision tools have been shown in primary care to be effective antimicrobial stewardship interventions.^36^ This highlights the potential benefit of these digital technologies to assist with improving prescribing, although adaptation of this tool to local guidelines and further research are needed to realise these potential benefits.

This study highlighted the lack of uniform drug resources for dental practitioners in Southeast Asia, with a range of resources (up to 62) being cited for use, with varying levels of evidence. Despite some countries, such as Indonesia, Malaysia, and Singapore,^12^, ^13^, ^14^ having guidelines for dentistry, dentists still use various resources for medicines information. These include unregulated and unreliable sources, such as Internet searches and artificial intelligence tools such as ChatGPT. This study further highlights the need to develop and/or reinforce the use of local guidelines and dental-specific drug compendia. Guidelines should be based on the availability and access to medicines and dental care, as well as local resistance patterns.^37^ Further research could be directed toward adapting MIMS Drugs4dent with local guidelines or from other appropriate resources, such as the WHO Aware Antibiotic book.

Features of MIMS Drugs4dent, such as information about the impact of drugs on dental treatment, were acknowledged by participating dentists that this would assist with safe prescribing and patient management. Digital medicine tools have been shown to reduce medication errors, optimise prescribing, and reduce exposure to unnecessary side effects and, as such, improve patient safety.^38^ Utilization and uptake are required to realise these benefits.^38^ This tool aligns with the recommendations from the WHO strategic action plan, the Bangkok declaration, and the ROADMAP for Southeast Asia to use digital technologies to improve oral care provision and patient safety.^3^^,^^4^^,^^15^ Further research can be directed toward understanding the barriers and enablers of provider attitudes toward such digital innovations.

This study has some limitations. First, while sufficient numbers of dental practitioners were recruited from each country, there was a predominance of practitioners working in the public sector. These practitioners may see more medically complex patients and thus be interested in a decision support tool. Second, the drugs in MIMS Drugs4dent are from an Australian compendium, so some medicines available in Southeast Asia are not listed. The dental guidance provided and the drug formulations are Australian. Both of these aspects were reflected in usability responses. These four countries vary in access to and availability of dental care, as well as digital technology infrastructure. The experiences of these dental practitioners will not be reflective of all practitioners in Southeast Asia. In addition, convenience sampling in this study resulted in predominantly public-sector practitioners, limiting generalisability. Further, the small sample size within subgroups restricted any further stratified analysis. Finally, MIMS Drugs4dent is in English, and while dentists in the four countries work in English, this will not be the case for all countries in the region. Nonetheless, to our knowledge, this is the first trial of a dental medicines tool in these countries. With its high acceptability, it has potential for adaptation for Southeast Asia. Further research can be directed toward adapting the tool using region-specific information, including a local drug formulary and guidelines. An exploration of the context in which these tools are used is needed for their effective implementation and uptake among more countries in Southeast Asia. After this study was completed, MIMS Drugs4dent was mobile rendered, which should assist with usability. With appropriate adaptation, MIMS Drugs4dent has the potential to transform access to timely, evidence-based drug information for dental practitioners in these countries.

## Conclusion

Dentists require access to e-health resources and infrastructure to support improved patient management with respect to medicines and prescribing. MIMS Drugs4dent demonstrated high rates of acceptability and usability in this study. Digital technologies can support dental practitioners in this region, and the study also highlights the importance of including dentistry in broader digital health strategies. MIMS Drugs4dent aligns with the WHO’s action plan for employing digital innovations to strengthen oral health care.

## Author contributions

L.T. was responsible for the conception and design of the study, interpretation of data, and drafting of the article. R.B. was responsible for the design of the study and analysis of the data. M.T. was responsible for data analysis and interpretation. W.T. was responsible for the design of the study. C.G. was responsible for the acquisition of data. J.C. was responsible for the acquisition of data. Y.S.W. was responsible for the acquisition of data. M.H.A. was responsible for the acquisition of data. W.N.W.A.K. was responsible for the acquisition of data. S.S. was responsible for the acquisition of data. B.I.N.A. was responsible for the acquisition of data. N.K. was responsible for data interpretation and analysis. M.M. was responsible for the design of the study and analysis of data. All authors revised the article critically for important intellectual content and gave final approval of the version to be submitted.

## Funding

L.T. was supported by a National Health and Medical Research Council Investigator Grant (2016647).

## Conflict of interest

L.T. and M.Mc. are recognised as inventors of MIMS Drugs4dent at the University of Melbourne. MIMS Drugs4dent is a commercial product, and L.T. and M.M. will receive royalties in the future.

## Appendix Supplementary materials

Image, application 1

## Footnotes

Supplementary material associated with this article can be found in the online version at doi:10.1016/j.identj.2025.109361.
