# Supplementary material for: Acceptability and Usability of a Digital Medicines Tool for Dental Practitioners in four Southeast Asian Countries
Source: Int Dent J. 2026 Jan 14;76(2):109361. doi: 10.1016/j.identj.2025.109361 (PMC12829107; doi:10.1016/j.identj.2025.109361)
Supplement: Supplementary file 1 [file mmc1.docx]

**Supplementary Table 1: Checklist for Reporting Of Survey Studies (CROSS)**

| **Section/topic** | **Item** | **Item description** | **Reported on page #** |
| --- | --- | --- | --- |
| **Title and abstract** | | |  |
| Title and abstract | 1a | State the word “survey” along with a commonly used term in title or abstract to introduce the study’s design. | 2 |
|  | 1b | Provide an informative summary in the abstract, covering background, objectives, methods, findings/results, interpretation/discussion, and conclusions. | 2 |
| **Introduction** | | |  |
| Background | 2 | Provide a background about the rationale of study, what has been previously done, and why this survey is needed. | 3-4 |
| Purpose/aim | 3 | Identify specific purposes, aims, goals, or objectives of the study. | 4 |
| **Methods** | | |  |
| Study design | 4 | Specify the study design in the methods section with a commonly used term (e.g., cross-sectional or longitudinal). | 4 |
|  | 5a | Describe the questionnaire (e.g., number of sections, number of questions, number and names of instruments used). | 5 |
| Data collection methods | 5b | Describe all questionnaire instruments that were used in the survey to measure particular concepts. Report target population, reported validity and reliability information, scoring/classification procedure, and reference links (if any). | 5 |
|  | 5c | Provide information on pretesting of the questionnaire, if performed (in the article or in an online supplement). Report the method of pretesting, number of times questionnaire was pre-tested, number and demographics of participants used for pretesting, and the level of similarity of demographics between pre-testing participants and sample population. | 5 |
|  | 5d | Questionnaire if possible, should be fully provided (in the article, or as appendices or as an online supplement). | Supplementary Figure 1 |
| Sample characteristics | 6a | Describe the study population (i.e., background, locations, eligibility criteria for participant inclusion in survey, exclusion criteria). | 5 |
|  | 6b | Describe the sampling techniques used (e.g., single stage or multistage sampling, simple random sampling, stratified sampling, cluster sampling, convenience sampling). Specify the locations of sample participants whenever clustered sampling was applied. | 4-5 |
|  | 6c | Provide information on sample size, along with details of sample size calculation. | 4 |
|  | 6d | Describe how representative the sample is of the study population (or target population if possible), particularly for population-based surveys. | 4 |
| Survey  administration | 7a | Provide information on modes of questionnaire administration, including the type and number of contacts, the location where the survey was conducted (e.g., outpatient room or by use of online tools, such as SurveyMonkey). | 5 |
|  | 7b | Provide information of survey’s time frame, such as periods of recruitment, exposure, and follow-up days. | 5 |
|  | 7c | Provide information on the entry process:  –>For non-web-based surveys, provide approaches to minimize human error in data entry.  –>For web-based surveys, provide approaches to prevent “multiple participation” of participants. | N/A  5 |
| Study preparation | 8 | Describe any preparation process before conducting the survey (e.g., interviewers’ training process, advertising the survey). | 5 |
| Ethical considerations | 9a | Provide information on ethical approval for the survey if obtained, including informed consent, institutional review board [IRB] approval, Helsinki declaration, and good clinical practice [GCP] declaration (as appropriate). | 5 |
|  | 9b | Provide information about survey anonymity and confidentiality and describe what mechanisms were used to protect unauthorized access. | 5 |
| Statistical  analysis | 10a | Describe statistical methods and analytical approach. Report the statistical software that was used for data analysis. | 6 |
|  | 10b | Report any modification of variables used in the analysis, along with reference (if available). | N/A |
|  | 10c | Report details about how missing data was handled. Include rate of missing items, missing data mechanism (i.e., missing completely at random [MCAR], missing at random [MAR] or missing not at random [MNAR]) and methods used to deal with missing data (e.g., multiple imputation). | N/A |
|  | 10d | State how non-response error was addressed. | N/A |
|  | 10e | For longitudinal surveys, state how loss to follow-up was addressed. | N/A |
|  | 10f | Indicate whether any methods such as weighting of items or propensity scores have been used to adjust for non-representativeness of the sample. | N/A |
|  | 10g | Describe any sensitivity analysis conducted. | N/A |
| **Results** | | |  |
| Respondent characteristics | 11a | Report numbers of individuals at each stage of the study. Consider using a flow diagram, if possible. | 7 |
|  | 11b | Provide reasons for non-participation at each stage, if possible. | N/A |
|  | 11c | Report response rate, present the definition of response rate or the formula used to calculate response rate. | 7 |
|  | 11d | Provide information to define how unique visitors are determined. Report number of unique visitors along with relevant proportions (e.g., view proportion, participation proportion, completion proportion). | N/A |
| Descriptive  results | 12 | Provide characteristics of study participants, as well as information on potential confounders and assessed outcomes. | 7 |
| Main findings | 13a | Give unadjusted estimates and, if applicable, confounder-adjusted estimates along with 95% confidence intervals and p-values. | N/A |
|  | 13b | For multivariable analysis, provide information on the model building process, model fit statistics, and model assumptions (as appropriate). | N/A |
|  | 13c | Provide details about any sensitivity analysis performed. If there are considerable amount of missing data, report sensitivity analyses comparing the results of complete cases with that of the imputed dataset (if possible). | N/A |
| **Discussion** | | |  |
| Limitations | 14 | Discuss the limitations of the study, considering sources of potential biases and imprecisions, such as non-representativeness of sample, study design, important uncontrolled confounders. | 12 |
| Interpretations | 15 | Give a cautious overall interpretation of results, based on potential biases and imprecisions and suggest areas for future research. | 10-12 |
| Generalizability | 16 | Discuss the external validity of the results. | 10-12 |
| **Other sections** | | |  |
| Role of funding source | 17 | State whether any funding organization has had any roles in the survey’s design, implementation, and analysis. | 7 |
| Conflict of interest | 18 | Declare any potential conflict of interest. | 12 |
| Acknowledgements | 19 | Provide names of organizations/persons that are acknowledged along with their contribution to the research. | 1 |

## **Supplementary Table 2: Reference information cited by participants as sources of drug information.**

| **Reference listed by participants** | **n** | **Freq (%)**  **N=120** |
| --- | --- | --- |
| Google Search | 46 | 38.3% |
| American Heart Association (AHA) guidelines | 43 | 35.8% |
| American Dental Association (ADA) Guidelines | 33 | 27.5% |
| MIMS | 19 | 15.8% |
| Formulari Ubat KKM (FUKKM) Blue Book Ministry of Health (MOH) Malaysia | 8 | 6.7% |
| American Association of Endodontics (AAE) Guidance on the Use of Systemic Antibiotics in Endodontics | 4 | 3.3% |
| National Antimicrobial Guidelines (NAG) | 4 | 3.3% |
| Rational Drug Use in Dentistry Faculty of Dentistry Chulalongkorn University | 4 | 3.3% |
| American Academy of Pediatric Dentistry (AAPD) Guidelines | 3 | 2.5% |
| American Association of Oral and Maxillofacial Surgeons (AAOMS) guideline | 2 | 1.7% |
| ChatGPT | 2 | 1.7% |
| National Antimicrobial Guidelines (NAG) Ministry of Health (MOH) Malaysia | 2 | 1.7% |
| NHGP guidelines | 2 | 1.7% |
| Textbook | 2 | 1.7% |
| Thai national formulary 2016 Drugs used in dentistry | 2 | 1.7% |
| American Association of Oral and Maxillofacial Surgeons’ Position Paper on Medication-Related Osteonecrosis of the Jaws—2022 Update | 1 | 0.8% |
| AMS | 1 | 0.8% |
| AMS Singapore guideline | 1 | 0.8% |
| Apps | 1 | 0.8% |
| Best practices | 1 | 0.8% |
| British Dental Journal (BDJ) | 1 | 0.8% |
| British National Formulary (BNF) | 1 | 0.8% |
| COLLEGE OF DENTAL SURGEONS SINGAPORE (CDSS) Clinical Practice Guidelines on the Use of Antibiotics in Dentistry Dec 2024 | 1 | 0.8% |
| CPG | 1 | 0.8% |
| CPG antibiotic prophylaxis in oral surgery for prevention of surgical site infection | 1 | 0.8% |
| CPG from ministry of health | 1 | 0.8% |
| Drug Doses (book by Frank Shann) | 1 | 0.8% |
| Drug registration Guidance Document-Malaysian NPRA | 1 | 0.8% |
| Drugs.com | 1 | 0.8% |
| EMC | 1 | 0.8% |
| ESE | 1 | 0.8% |
| Faculty of General Dental Practice (FGDP) UK | 1 | 0.8% |
| General Guidelines of Antibiotic Use" (MOH Decree No. 2406/2011) Indonesia | 1 | 0.8% |
| Google scholar | 1 | 0.8% |
| Guidelines by polyclinic | 1 | 0.8% |
| IE prophylaxis | 1 | 0.8% |
| Infectious Diseases Society of America (IDSA) | 1 | 0.8% |
| Lexi Comp online | 1 | 0.8% |
| Lexi-Comp Drug Information | 1 | 0.8% |
| Medscape | 1 | 0.8% |
| Micromedex 2.0 Online | 1 | 0.8% |
| MIMS Drugs4dent | 1 | 0.8% |
| MOH Guidelines | 1 | 0.8% |
| National University Polyclinic prescription guidelines and dental drug formulary | 1 | 0.8% |
| NHS Guidelines | 1 | 0.8% |
| NUHS Intranet | 1 | 0.8% |
| Old pharmaco notes from undergrad | 1 | 0.8% |
| Oral Medicine Books | 1 | 0.8% |
| PDAA guidelines | 1 | 0.8% |
| PDGI guidelines | 1 | 0.8% |
| Physician memo | 1 | 0.8% |
| Polyclinic guidelines | 1 | 0.8% |
| PPK | 1 | 0.8% |
| Previous diagnosis | 1 | 0.8% |
| pubmed. | 1 | 0.8% |
| RCS | 1 | 0.8% |
| Recent scientific journal reviews | 1 | 0.8% |
| Recommendations from colleagues | 1 | 0.8% |
| Sanford Antimicrobial Therapy | 1 | 0.8% |
| Sanford guidelines | 1 | 0.8% |
| School notes | 1 | 0.8% |
| SDCEP 2018 | 1 | 0.8% |
| Textbook of Dental Pharmacology | 1 | 0.8% |
| WebMD | 1 | 0.8% |
| WHO pain ladder | 1 | 0.8% |
| World Health Organization (WHO) AWaRe Antibiotic Book | 1 | 0.8% |
| **Grand Total** | **227** |  |

More than one reference was cited by participants

**Supplementary Figure 1**

**Acceptability and usability questionnaire**

MIMS Drugs4dent® is a novel dental clinical decision tool, aimed to provide dental-relevant information such as dental procedural considerations, oral adverse effects, and patient education about appropriate antibiotic use. It also provides drug and allergy interactions, information about drugs in pregnancy and lactation, and paediatric dose calculations for dentistry.

Thank you for your time in using the tool and providing us feedback on your thoughts and opinion of the tool for use in dental clinical practice. It will take approximately 10 minutes to complete.

**Demographics**

Q1. Which of the following best describes you?

- Male
- Female
- Prefer not to say

Q2. How many years of clinical experience do you have?

- <10 years
- 11-20 years
- 21-30 years
- >30 years

Q3. What is the postal code of your work location?

Q4. Where do you work most of the time?

- Private dental practice
- Public dental practice

Q5. What guidelines/resources do you currently use to guide your prescribing of antibiotics and pain relief?

[Free text]

**Part 1: About MIMS Drugs4dent^®^**

**Search for medications**

Q1. When searching for your patient’s current medications in this section:


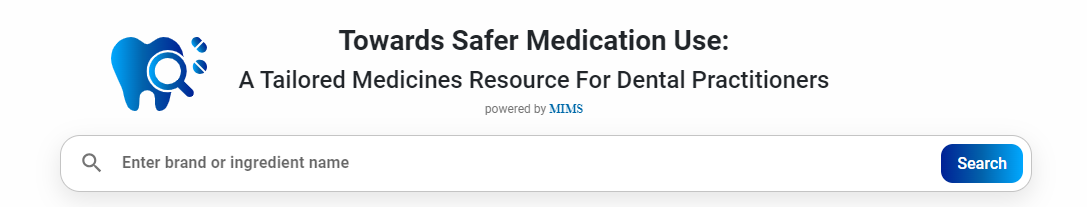


We have included information specific for dentistry, as well as dental procedural considerations and oral adverse effects. Is there other information you would like to know that might help you in clinical practice dentistry?

1. No, there is enough information needed for dental clinical practice
2. Yes, I would like more information about drugs

Q2. If yes, please indicate what other information you think should be included below:

[free text]

**Drug and allergy interactions**

Q3. Is this drug and allergy checker useful for dental clinical practice when prescribing medications?

a) Yes

b) No – If no, please explain why: [free text]

**Pregnancy and lactation**

Q4. Is the pregnancy and lactation information useful for dental clinical practice when prescribing medications?

a) Yes

b) No – If no, please explain why: [free text]

**Dental antibiotic use guide**

Q5. The section on Prescribing Guidelines was aimed to help assist dentists by providing patients education about appropriate antibiotic use for specific dental clinical scenarios, especially for those who expect or request antibiotics instead of treatment.

Is the Dental Antibiotic Use guide helpful for dental clinical practice when prescribing medications?

a) Yes

b) No – If no, please explain why: [free text]

**Dose calculations**

Q6. The section called “Dosing calculations” is aimed to help dentists prescribe according to Australian dental guidance, as well as assist with prescribing for children.

Do you find the dose calculator section helpful for dental clinical practice for paediatric prescribing? You can select more than one option.

1. Yes, I found this helpful and would use this as it is with no changes
2. Yes, but I would prefer the **dose calculations** to be from other guidelines (not Australian guidance)
   1. Please list the guidelines: [Free text]
3. Yes, but the **dose formulations** are different to what we have available in Singapore; I would prefer the doses provided in formulations that are available in Singapore
4. No, I do not find this helpful – If no, please explain why: [free text]

**Part 2: Acceptability of MIMS Drugs4dent^®^**

Q7. How comfortable did you feel using Drugs4dent^®^?

1) Very uncomfortable

2) Uncomfortable

3) Neither uncomfortable or comfortable

4) Comfortable

5) Very comfortable

Q8. How much effort did it take to use Drugs4dent^®^?

1) No effort at all

2) A little effort

3) Neither little effort or a lot

4) A lot of effort

5) Huge effort

[Questions 9-13 will be in a table]

Q9. Drugs4dent^®^ can improve my ability to access dental-relevant drug knowledge (e.g. which drugs cause medication-related osteonecrosis of the jaw, increased bleeding risk etc) to assist with improved patient management regarding medication use.

1) Strongly disagree

2) Disagree

3) Neither agree or disagree

4) Agree

5) Strongly agree

Q10. Drugs4dent^®^ can improve my ability to prescribe appropriately, regarding correct regimen (dose, duration and frequency) according to guidelines.

1) Strongly disagree

2) Disagree

3) Neither agree or disagree

4) Agree

5) Strongly agree

Q11. Drugs4dent^®^ can improve my ability to prescribe medicines appropriately and safely, with respect to drug and allergy interactions, pregnancy and breastfeeding considerations.

1) Strongly disagree

2) Disagree

3) Neither agree or disagree

4) Agree

5) Strongly agree

Q12. It is clear to me how Drugs4dent^®^ will help me access dental-relevant drug knowledge and prescribe more safely.

1) Strongly disagree

2) Disagree

3) Neither agree or disagree

4) Agree

5) Strongly agree

Q13. Engaging with Drugs4dent^®^ chairside would interfere with my work flow.

1) Strongly disagree

2) Disagree

3) Neither agree or disagree

4) Agree

5) Strongly agree

Q14. How confident did you feel about using Drugs4dent^®^?

1) Very unconfident

2) Unconfident

3) Neither confident or unconfident

4) Confident

5) Very confident

Q15. How acceptable was MIMS Drugs4dent^®^?

1) Completely unacceptable

2) Unacceptable

3) Neither acceptable or unacceptable

4) Acceptable

5) Completely acceptable

**Part 3: Usability of MIMS Drugs4dent^®^**

[Questions 16 to 22 will be in a table]

Q16. Overall I found it easy it is to use MIMS Drugs4dent^®^

1) Strongly disagree

2) Disagree

3) Neither agree or disagree

4) Agree

5) Strongly agree

Q17. I was able to access information I need quickly using MIMS Drugs4dent^®^

1) Strongly disagree

2) Disagree

3) Neither agree or disagree

4) Agree

5) Strongly agree

Q18. It was easy to learn to use MIMS Drugs4dent^®^

1) Strongly disagree

2) Disagree

3) Neither agree or disagree

4) Agree

5) Strongly agree

Q19. Whenever I made a mistake using MIMS Drugs4dent^®^, I could recover easily and quickly.

1) Strongly disagree

2) Disagree

3) Neither agree or disagree

4) Agree

5) Strongly agree

Q20. It was easy for me to find the information I needed in MIMS Drugs4dent^®^

1) Strongly disagree

2) Disagree

3) Neither agree or disagree

4) Agree

5) Strongly agree

Q21. The interface of MIMS Drugs4dent^®^ was pleasant.

1) Strongly disagree

2) Disagree

3) Neither agree or disagree

4) Agree

5) Strongly agree

Q22. MIMS Drugs4dent^®^ has all the functions and capabilities I need for accessing drug knowledge and appropriate prescribing.

1) Strongly disagree

2) Disagree

3) Neither agree or disagree

4) Agree

5) Strongly agree

Questions 23-26 will be presented in a table

Q23. Overall, I am satisfied with MIMS Drugs4dent^®^.

1) Strongly disagree

2) Disagree

3) Neither agree or disagree

4) Agree

5) Strongly agree

Q24. MIMS Drugs4dent^®^ is an important and helpful tool in clinical practice, to help with safe prescribing and appropriate use of medicines in dentistry.

1) Strongly disagree

2) Disagree

3) Neither agree or disagree

4) Agree

5) Strongly agree

Q25. I would like to have MIMS Drugs4dent^®^ available to me for clinical practice dentistry.

1) Strongly disagree

2) Disagree

3) Neither agree or disagree

4) Agree

5) Strongly agree

**Part 4: Clinical workflow and general comments about content of MIMS Drugs4dent®**

Q26. The clinical workflow using Drugs4dent^®^ was clear.

1) Strongly disagree

2) Disagree

3) Neither agree or disagree

4) Agree

5) Strongly agree

Q27. Now that you have used MIMS Drugs4dent^®^, how often do you anticipate that you would use this tool?

1) Once <5 patients

2) Once every 5-10 patients

3) Once every 10-20 patients

4) Once >20 patients

Q28. Would you prefer the tool integrated into your dental practice software (linked to individual patients, with patient information and prescriptions stored in your dental practice management software), or do you prefer it as a standalone system?

1. Yes, I think integration would be better
2. No, I prefer Drugs4dent^®^ as a standalone system

Q29. Is there any other information you think should be included in MIMS Drugs4dent^®^?

[Free text]

Q30. Is there any other feedback you can give us about MIMS Drugs4dent^®^?

[Free text]
